# Prospective evaluation of medication-related problems and pharmacist interventions in liver transplant recipients

**DOI:** 10.3389/fphar.2026.1738563

**Published:** 2026-02-23

**Authors:** Sena Güzel Karahan, Mefküre Durmuş, Ahmet Çakır, Zeynep Ülkü Gün, Sezai Yılmaz

**Affiliations:** 1 Department of Clinical Pharmacy, Faculty of Pharmacy, Inonu University, Malatya, Türkiye; 2 Department of Clinical Pharmacy, Faculty of Pharmacy, Karadeniz Technical University, Trabzon, Türkiye; 3 Department of Clinical Pharmacy, Faculty of Pharmacy, Trakya University, Edirne, Türkiye; 4 Department of General Surgery, Faculty of Medicine, Inonu University, Malatya, Türkiye

**Keywords:** clinical pharmacy, intensive care, liver transplant, medication-related problems, Pharmaceutical Care Network Europe

## Abstract

**Background:**

Medication-related problems (MRPs) are a common patient safety issue among hospitalized individuals, often associated with reduced quality of life, increased healthcare costs, and higher mortality. Due to the chronic and complex nature of post-transplant care, liver transplant recipients are particularly vulnerable to MRPs. Clinical pharmacists play a critical role in identifying and resolving MRPs, thereby promoting the rational use of medications. The objective of this study was to characterize MRPs among liver transplant recipients and assess the clinical determinants associated with their occurrence.

**Method:**

This prospective study was conducted between 5 October 2023 to 31 April 2024 at the Liver Transplantation Institute. A total of 373 hospitalized liver transplant recipients in inpatient wards and intensive care units who were receiving at least one medication were included. Donors and patients not receiving any medication were excluded. The Pharmaceutical Care Network Europe (PCNE) classification system version 9.1 was used to categorize MRPs. Clinical and demographic characteristics of patients with and without MRPs were compared statistically and risk factors were analyzed through logistic regression.

**Results:**

Among the 373 patients included in this study, at least one MRP was identified in 311 patients, yielding 620 MRPs in total. A total of 620 interventions were proposed, of which 547 (88.2%) were accepted, while 73 (11.8%) were rejected. The leading causes of MRPs was “dose selection” (C3) was the most common cause (44.2%), followed by “drug selection” (C1) at 26.1%. The presence of at least one comorbidity and acute kidney injury were significant risk factors for the occurrence of MRPs (p < 0.05).

**Conclusion:**

To our knowledge, this is the first and most comprehensive study applying the PCNE v.9.1 method to liver transplant recipients hospitalized in both ward and intensive care unit settings. The most prevalent MRPs were related to “treatment effectiveness,” primarily caused by “dose selection” and “drug selection.” Clinical pharmacists and physicians should particularly focus on these aspects when reviewing transplant patients’ medication regimens. The results achieved in this study suggest that clinicians should exercise caution when prescribing new medications to transplant recipients with at least one comorbidity and a history of acute kidney injury.

## 1 Introduction

Since its first implementation by Starzl et al., in 1967, liver transplantation has become the gold standard treatment for various liver diseases, including cirrhosis, fulminant hepatitis, certain metabolic liver disorders, and primary or metastatic liver tumors (Abbasoglu, 2008; Eris et al., 2013). In Türkiye, the history of transplantation began with Dr. Mehmet Haberal. In 1988, he performed the first adult-to-adult deceased-donor liver transplant in Türkiye, followed by the first adult-to-child living-donor liver transplant in 1990 (Akbulut et al., 2015). Liver transplant recipients require lifelong immunosuppressive therapy to prevent graft rejection and ensure the success of solid organ transplantation (SOT). Achieving therapeutic goals typically necessitates careful monitoring and individualized dosing of immunosuppressive agents. In addition to the inherent complexity of immunosuppressive pharmacotherapy, many other pharmacotherapeutic issues must be considered in transplant recipients (Lauchart et al., 2005).

The Pharmaceutical Care Network Europe (PCNE) defines any event or circumstance that interferes with achieving the desired outcomes of therapy as a medication-related problem (MRP) (Van Mil et al., 2004). MRPs lead to increased patient morbidity and mortality, higher rates of hospital admissions, prolonged hospital stays, and, consequently, elevated healthcare costs (Jung-Poppe et al., 2022). Since many MRPs are preventable, identifying specific risk factors that predispose patients to their occurrence is of critical importance for optimizing healthcare quality, resource utilization, and patient safety (Saldanha et al., 2020). Risk factors for MRPs in this patient population include polypharmacy, adverse drug events, drug-drug interactions, and the inability to maintain immunosuppressive drugs within their optimal therapeutic range. Rational, safe, and cost-effective pharmacotherapy relies not only on accurate diagnosis and effective treatment monitoring but also on the patient’s adherence to the prescribed regimen (Maxwell, 2016).

Clinical pharmacist interventions play a key role in each of these aspects by ensuring the safe and effective use of medications. Numerous studies have demonstrated the crucial role of clinical pharmacists in identifying and resolving MRPs in hospital settings (Adusumıllı and Adepu, 2014). Given the complexity of post-transplant management, clinical pharmacists, who have specialized training in pharmacotherapy, are recognized as integral members of multidisciplinary transplant teams, as defined by the United Network for Organ Sharing (UNOS) (Ah et al., 2016). However, in order to systematically develop documents, and track pharmaceutical care practices, clinical pharmacists need a robust classification system. The structure and functionality of such a classification system are also essential. The PCNE classification system categorizes MRPs under five main domains: problems, causes, planned interventions, acceptance of interventions, and the status of the MRP. This structure allows for a comprehensive classification from the identification of MRPs to the implementation of solutions (Van Mil et al., 2020; Satria et al., 2022).

This study aims to highlight the contribution of clinical pharmacists through the provision of pharmaceutical care services to patients hospitalized in the liver transplant unit of a university hospital, identification of MRPs using the PCNE classification system, and the verbal communication of proposed solutions to the attending physician and/or other healthcare personnel.

## 2 Methods

This study was carried out at the Liver Transplant Institute of a university hospital between 5 October 2023, and 31 April 2024. Both ICU and ward patients hospitalized at the Liver Transplant Institute were included. Inclusion criteria were being hospitalized at the Liver Transplant Institute, a minimum hospital stay of at least 24 h, being a liver transplant recipient, being on at least one medication, and being assessed by a clinical pharmacist. The exclusion criteria were being a liver donor, not being a liver transplant recipient, length of stay shorter than 24 h, refusing to sign or not signing the informed consent form, not taking any medication, and being admitted and discharged on days when the clinical pharmacist was not present. Patients who voluntarily withdrew from the study during the course of the research (either by themselves or with the consent of their legal guardians) were excluded. During the study period, a total of 610 patients were admitted to the liver transplantation institute. Of these, 207 patients were excluded because they were donors, 2 were excluded due to absence of medication use, and 28 were excluded as they were admitted and discharged on days when a clinical pharmacist was unavailable. A total of 373 patients meeting the specified criteria were included in this study. Informed consent was obtained from all individual participants or their legal guardians.

Ethical Approval: Ethical approval for this study was obtained from the Non-Interventional Clinical Research Ethics Committee of Inonu University on 4 October 2023 (Decision No: 2023\5026).

The study was designed as a prospective observational investigation, employing both descriptive statistical methods and analytical analyses to evaluate the correlations with selected factors concerning MRP. MRPs were initially assessed using descriptive approaches, followed by comparisons between patients with and without MRPs according to clinically relevant variables such as comorbidity profiles, admission causes, care settings, and medication classes. Analyses were performed at the patient level, and multiple MRPs within a single patient were not considered independent observations.

Patients were divided into two groups regarding the analyzing methods: The groups included those with MRPs and those without. In addition, two subgroups were created to examine differences between patients in the ICU and those in the general ward. The clinical and demographic characteristics of patients in each group were recorded. The medical treatments administered during hospitalization were assessed daily. MRPs observed in the medical treatment of patients by a clinical pharmacist enrolled in a clinical pharmacy residency program were prospectively evaluated. The clinical pharmacist participated in daily ward rounds with the clinical team, during which identified MRPs were presented and discussed. Recommendations regarding the MRPs were shared with the responsible physician and/or other healthcare professionals. The acceptance or rejection of these recommendations, along with the reasons for any rejections, was documented. The resolution status of each MRP was also followed by the clinical pharmacist. The PCNE classification system v9.1, which is an internationally recognized and previously validated framework for the classification of MRPs, was used in this study. The officially available Turkish version of PCNE v9.1 classification was applied in accordance with the PCNE methodological guidance. Problems that did not fit into the predefined categories of the PCNE system were classified as “unspecified” or “other” problems.

### 2.1 Assessment

Patient profiles were created using demographic characteristics, laboratory data, comorbidities, and medication-related information collected before and during hospitalization. MRPs were identified through systematic medication review, medication reconciliation at admission, therapeutic drug monitoring, and assessment of drug selection and dosing. Routine clinical pharmacy services, including patient counseling and medication education, were provided as part of standard clinical care.

Pediatric patients were defined as those who were less than 18 years of age. Polypharmacy was defined as the concomitant use of five or medications (Masnoon et al., 2017). Patients estimated glomerular filtration rates were calculated using the 2021 Chronic Kidney Disease Epidemiology Collaboration (CKD-EPI) formula (Lee et al., 2025). Chronic kidney disease (CKD) was defined as structural or functional kidney abnormalities persisting for at least 3 months (Stevens et al., 2024). CKD stages, acute kidney injury (AKI), and renal replacement therapy were all recorded. Drug selection, dose regimens, and potential drug-drug interactions were evaluated based on national and international clinical guidelines using UpToDate ® and Lexicomp ®. All identified MRPs and related recommendations were communicated to the responsible physician or healthcare staff. MRPs, interventions, and outcomes were classified using the PCNE v9.1 system (Van Mil et al., 2020).

### 2.2 Statistical analysis

The statistical analysis of the data was performed using IBM SPSS Statistics (Statistical Package for the Social Sciences) version 27.0. The study was designed as a prospective observational inquiry to include all eligible liver transplant patients admitted within a specified timeframe, rather than as a hypothesis-driven study with a preset sample size. Thus, the study population consisted of all patients who met the inclusion criteria during the study period, forming a consecutive cohort representative of routine clinical practice. The normality of quantitative data was assessed using the Kolmogorov-Smirnov test. As the data did not conform to a normal distribution, continuous variables were summarized as medians. Quantitative data from two groups were compared using the Mann-Whitney U test, while comparisons across more than two groups were conducted using the Kruskal-Wallis H test. The chi-square test was applied for comparisons of categorical variables, and relationships between qualitative variables in two groups were also assessed using the chi-square test. Multivariate analysis was conducted using logistic regression. Variables included in the multivariable logistic regression model were those showing a statistically significant association at the level by 0.2 with MRPs in univariable analyses (p < 0.2). Given the limited number of events, a highly parsimonious model was intentionally constructed to minimize overfitting and model instability. The variance explained by the model was indicated by the Nagelkerke R^2^ value, and model fit was evaluated using the Hosmer-Lemeshow test. Risk factors were presented as odds ratios (OR). A p-value of <0.05 was considered statistically significant.

## 3 Results

373 patients were included in this study, consisting of 141 females (37.8%) and 232 males (62.2%). The median age of all patients was 50 years, with an interquartile range (IQR) of 35–61. The median age was 49 years (IQR: 26.5–60) for females and 51.5 years (IQR: 37.25–61) for males (Table 1). 51 (13.7%) patients were pediatric patients whose age were less than 18 years. The overall mortality rate was 25.5% (n = 96, n = 9 pediatric and n = 87 adult patients).

**TABLE 1:** Univariable and multivariable analyses of demographic characteristics according to medication related problems.

​	Total (%)	Univariate analysis			Multivariate analysis	
		With MRP (%)	No MRP (%)	*p*	OR (CI)	*P^*
Sex						
Male	232 (62.2)	194 (52,0)	38 (10,2)	0.887*	-	*-*
Female	141 (37.8)	117 (31,36)	24 (6,44)	​	-	*-*
Patient age group						
Adult	322 (86.3)	265 (71,02)	57 (15,28)	0.159*	-	0.225
Pediatric	51 (13.7)	46 (12,36)	5 (1,34)	​	​	​
Median age (IQR)	50 (35–61)	52 (34–61)	47 (36–58)	0.431**	-	*-*

*Pearson Chi-square test **Fischer Exact Test ^Logistic regression.

Since all included patients were taking five or more medications, polypharmacy was identified in the entire study population. The median number of medications used per patient was 15 (IQR: 12–19).

Both general ward and intensive care unit (ICU) patients were included in the study. Of the total cohort, 194 patients (52%) were admitted to the ICU and 179 patients (48%) to the general ward. Patient information is given in Table 2. Pediatric patients 27 (7.2%), and adult patients, 167 (44.8%) were admitted to the ICU. There was no relationship between pediatric or adult patients and admission units (*p* < 0.05).

**TABLE 2:** Univariable and multivariable analyses of medication related problem occurrence according to clinical care settin**g**.

​	Total (%)	Univariate analysis			Multivariate analysis	
		With MRP (%)	No MRP (%)	*p*	OR (CI)	*P^*
Hospital unit						
ICU	194 (52.01)	166 (44,50)	28 (7,51)	0.237*	-	*-*
Ward	179 (47.99)	145 (38,87)	34 (9,12)	​	​	​
Median number of medications (IQR)	15 (12–19)	16 (12–20)	13.5 (8–27)	<0.001**	-	0.196
ICU	194 (52.01)	16 (13–20)	13 (11–15.75)	0.358**	-	*-*
Ward	179 (47.98)	15 (12–19)	14 (11–15.25)	​	-	*-*

*Pearson Chi-square test **Fischer Exact Test t ^Logistic regression.

Statistically significant values are indicated in bold in the relevant tables (*p* < 0.05).

The most common reasons for hospitalization were hepatitis B-related cirrhosis (21.4%) and cryptogenic cirrhosis (19%). Patient information is given in Table 3. Comorbidities were identified in 312 patients (83.6%), with hypertension being the most prevalent, followed by diabetes mellitus. Patient information is given in Table 4.

**TABLE 3:** Univariable analyses of medication related problem occurrence according to hospital admission indication.

Cause of hospital admission	Total (%)	Univariate analysis		
		With MRP (%)	No MRP (%)	*p*
Hepatitis-B-related cirrhosis	81 (21.7)	71 (19.0)	10 (2.7)	0.355*
Cryptogenic cirrhosis	71 (19)	60 (16.1)	11 (2,9)	
HCC	29 (7.8)	24 (6.4)	5 (1.4)	
Autoimmune hepatitis	27 (7.2)	21 (5.6)	6 (1.6)	
Acute fulminant hepatitis	25 (6.7)	24 (6,5)	1 (0.2)	
Wilson disease	20 (5.4)	17 (4.6)	3 (0.8)	
Hepatitis-C-related cirrhosis	17 (4.6)	14 (3.8)	3 (0.8)	
Budd Chiari	17 (4.6)	15 (4.0)	2 (0.6)	
Primary sclerosing cholangitis	17 (4.6)	14 (3.8)	3 (0.8)	
HBV + HDV	16 (4.3)	12 (3.2)	4 (1.1)	
Biliary atresia	12 (3.2)	8 (2.1)	4 (1.1)	
Ethanol-related cirrhosis	11 (2.9)	8 (2.1)	3 (0.8)	
PFIC	10 (2.7)	10 (2.7)	0	
NASH	3 (0.8)	2 (0.6)	1 (0.2)	
Primary biliary cirrhosis	3 (0.8)	2 (0.6)	1 (0.2)	
Portal vein thrombosis	2 (0.6)	2 (0.6)	0	
GI bleeding	1 (0.2)	1 (0.2)	0	

*Pearson Chi-square test **Fischer Exact Test *** Mann Whitney U test ^Logistic regression. GI: gastrointestinal; HCC: hepatocellular carcinoma; HBV: Hepatitis B virus; HDV: Hepatitis D virus; NASH: nonalcoholic steatohepatitis; PFIC: progressive familial intrahepatic cholestasis.

**TABLE 4:** Univariable and multivariable analyses of medication related problem occurrence according to comorbidity.

​	Total (%)	Univariate analysis			Multivariate analysis	
		With MRP (%)	No MRP (%)	*p*	OR (CI)	*P^*
Comorbidity	312 (83.6)	269 (72.11)	43 (11.5)	0.001*	**2.69 (1.11–6.5)**	**0.028**
Hypertension	169 (45.3)	144 (38.6)	25 (6.7)	0.388*	-	-
Diabetes	149 (39.9)	116 (31.1)	33 (8.8)	0.019*	**0.323 (0.154 -0.680)**	**0.003**
Hyperlipidemia	36 (9.7)	33 (8.8)	3 (0.9)	0.160*	-	0.108
Thyroid disorders					-	-
Hypothyroidism	13 (3.5)	10 (2.6)	3 (0.9)	0.360*	-	-
Hyperthyroidism	2 (0.6)	1 (0.3)	1 (0.3)		-	-
Chronic kidney disease						
G1	62 (16.6)	44 (11.8)^a^	18 (4.8)	0.008*	-	0.976
G2	21 (5.6)	15 (4.0)	6 (1.6)			
G3a	16 (4.3)	15 (4.0)	1 (0.3)			
G3b	20 (5.4)	20 (5.4)^a^	0			
G4	7 (1.9)	7 (1.9)	0			
G5	8 (2.1)	8 (2.1)	0			
AKI on CKD	25 (6.7)	25 (6.7)	0	0.012**	-	0.998
AKI	85 (22.8)	84 (22.5)	1 (0.3)	<0.001*	**16.453 (1.72–156.9)**	**0.015**
RRT					-	-
Hemodialysis	12 (3.2)	11 (2.9)	1 (0.3)	<0.001*	-	0.777
SRRT	71 (20.7)	71 (20.7)	0			
Neurological diseases					-	-
Epilepsy	18 (4.8)	14 (3.8)	4 (1.0)	0.910*	-	-
Parkinson’s disease	8 (2.1)	7 (1.8)	1 (0.3)		-	-
CVD	7 (1.8)	6 (1.5)	1 (0.3)		-	-
Respiratory disease					-	-
Asthma	63 (16.9)	50 (13.4)	13 (3.5)	0.588*	-	-
COPD	27 (7.2)	22 (5.9)	5 (1.3)		-	-
Pulmonary embolism	5 (1.3)	5 (1.3)	0		-	-
Cardiovascular disease					-	-
AF	69 (18.5)	60 (16.1)	9 (2.4)	0.459*	-	-
Heart failure	4 (1.1)	4 (1.1)	0		-	-
Atherosclerotic heart disease	27 (7.2)	24 (6.4)	3 (0.8)		-	-

*Pearson Chi-square test **Fischer Exact Test *** Mann Whitney U test ^Logistic regression. GI, gastrointestinal; COPD, chronic obstructive pulmonary disease; AKI, acute kidney injury; CKD, chronic kidney disease; RRT, renal replacement therapy; SRRT, sustained renal replacement therapy; AF, atrial fibrillation; CVD, cerebrovascular disease.

^a^ Statistically significant difference within the same category.

Statistically significant values are indicated in bold in the relevant tables (*p* < 0.05).

Since all patients in this study were liver transplant recipients, the immunosuppressive therapy was administered to all. Apart from immunosuppressants, the most frequently used drug classes were proton pump inhibitors (99.5%) and antimicrobial agents (99.5%). The distribution of drugs used in patients according to the presence of MRP is given in Table 5. Spearman correlation analysis in the group with MRPs revealed a positive correlation between the number of MRPs and the number of antimicrobial agents (r = 0.175, *p* = 0.001) and antihypertensive drugs (r = 0.133, *p* = 0.01), indicating that MRP frequency increased with the number of these medications.

**TABLE 5:** Distribution of medications used in patients by the presence of MRPs.

Medication group	Frequency (%)	With MRP (%)	Without MRP (%)	*p*
Antimicrobial agents	371 (99.5)	309 (99.4)	62 (100)	1**
Antidiabetic agents	138 (37)	106 (34.1)	32 (51.6)	0.009*
Antihypertensive agents	179 (48)	156 (50.2)	23 (37.1)	0.06*
Proton pump inhibitors	371 (99.5)	310 (99.7)	61 (98.4)	0.305**
Immunosuppressive agents	373 (100)	311 (100)	62 (100)	-
Monotherapy	120 (32.2)	98 (31.5)	22 (35.5)	0.541*
Dual therapy	151 (40.5)	126 (40.5)	25 (40.3)	0.978*
Triple therapy	111 (29.8)	94 (30.2)	17 (27.4)	0.659*

*indicates the Pearson Chi-Square test.

**indicates the Fisher’s Exact test.

Statistically significant values are indicated in bold in the relevant tables (*p* < 0.05).

Throughout the study period, a total of 620 suggestions were made for 373 patients. At least one MRP was identified in 311 patients (83.37%). The mean number of MRPs per patient was calculated to be 1.66. The total number of medications was significantly higher in patients with MRPs (median 16, IQR: 12–20) compared to those without MRPs (median 13.5, IQR: 8–27) (*p* < 0.001). No significant difference was found between ICU and general ward patients in terms of the presence of at least one MRP (*p* > 0.05). Additionally, there was no relationship between pediatric and adult patients in terms of the presence of at least one MRP (*p* > 0.05). However, when evaluating the total number of MRPs, ICU patients had a significantly higher number of MRPs per patient (*p* < 0.05). The presence of comorbidities, acute kidney injury (AKI), continuous renal replacement therapy (CRRT), acute-on-chronic kidney injury, and stage G3b chronic kidney disease (CKD) were significantly associated with MRP occurrence (p < 0.05). Having stage G1 of CKD was found to be associated with the absence of MRPs (Table 4).

In this study, medication dosing problems among patients with impaired renal function were systematically assessed based on their hospital unit. Issues related with renal dose adjustment were identified in 24.3% of ICU patients and 22.4% of general ward patients.

Factors contributing to elevated MRP risk were identified by evaluating both the total number of medications and clinical characteristics. Multicollinearity among independent variables was assessed using variance inflation factors (VIF). All VIF values were below 2, indicating no significant multicollinearity. Model calibration was assessed using the Hosmer–Lemeshow goodness-of-fit test, which indicated adequate fit (*p* = 0.768). The presence of at least one comorbidity (OR: 2.69, CI:1.11–6.5) and the occurrence of acute kidney injury (OR: 16.4, CI: 1.7–156.9) were found to be independent risk factors for MRPs (Table 4).

Among the classified problems, “treatment effectiveness” (P1) was the most frequently observed category, accounting for 59.8% of all MRPs. Regarding causes of MRPs, the most frequent category was “dose selection” (C3), observed in 44.2% of cases. Most pharmacist interventions were categorized as “at the drug level” (I3), accounting for 43.45% of all interventions. Of these, 88.2% were accepted by the clinical team. The resolution status of MRPs following the interventions is presented in Table 6. Supplementary Table S1 provides representative, real-word clinical examples of MRPs from our cohort.

**TABLE 6:** Classification of MRPs and planned interventions.

Type of MRP	Total n (%)	Ward n (%)	ICU n (%)	Pediatric patients n (%)	Adult patients n (%)
P1. Treatment effectiveness	371 (59.8)	132 (21.3)	239 (38,5)	54 (15)	317 (85)
P1.2. Drug therapy not sufficiently effective	220 (35.5)	81 (13.1)	139 (22.4)	39 (17.8)	181 (82.2)
P1.3. There are untreated symptoms or indications	151 (24.3)	51 (8.2)	100 (16.1)	15 (10)	136 (90)
P2.1. Treatment safety	213 (34.4)	69 (11.1)	144 (23.3)	30 (14)	183 (86)
P3. Others	36 (5.8)	17 (2.7)	19 (3.1)	6 (16.7)	30 (83.3)
P3.1. Unnecessary drug therapy	36 (5.8)	17 (2.7)	19 (3.1)	6 (16.7)	30 (83.3)
P. Total	620 (100)	218 (35.1)	402 (64.9)	90 (14.5)	530 (85.5)
Causes of MRPs					
C1 drug selection	237 (26.1)	78 (8.6)	159 (17.5)	23 (9.7)	214 (90.3)
C1.1. Drug not appropriate per guidelines/formulary	26 (2.9)	10 (1.1)	16 (1.8)	3 (11.5)	23 (88.5)
C1.2. No indication for the drug	15 (1.7)	8 (0.9)	7 (0.8)	1 (6.7)	14 (93.3)
C1.3. Inappropriate combination with other drugs and/or supplements such as herbal ones	19 (2,1)	5 (0.6)	14 (1.5)	1 (5.3)	18 (94,7)
C1.4. Unnecessary duplication within drug class or active ingredient	13 (1.4)	5 (0.5)	8 (0.9)	2 (15.4)	11 (84.6)
C1.5. Drug not prescribed for an existing indication	162 (17.9)	50 (5.6)	112 (12.3)	15 (9.3)	147 (90.7)
C1.6 Excessive variety of drugs for same indication	2 (0.2)	0 (0)	2 (0.2)	1 (50)	1 (50)
C2 Dosage form	37 (4.3)	15 (1.8)	22 (2.5)	6 (16.2)	31 (83.8)
C2.1. Inappropriate dosage form/formulation for the patient	37 (4.3)	15 (1.8)	22 (2.5)	6 (16.2)	31 (83.8)
C3. Dosage selection	399 (44.0)	129 (14.2)	270 (29.8)	85 (21.3)	314 (78.7)
C3.1. Drug dose too low	73 (8.0)	24 (2.6)	49 (5.4)	18 (24.7)	55 (75.3)
C3.2. Drug dose too high	166 (18.3)	53 (5.8)	113 (12.5)	34 (20,5)	132 (79,5)
C3.3. Dosing regimen not frequent enough	33 (3.7)	8 (0.9)	25 (2.8)	6 (18.2)	27 (81.8)
C3.4. Dosing regimen too frequent	124 (13.7)	42 (4.7)	82 (9)	26 (21)	98 (79)
C3.5. Instructions regarding timing of dose are unclear, incorrect, or incomplete	3 (0.3)	2 (0.2)	1 (0.1)	1 (33.3)	2 (66.7)
C4. Duration of therapy	24 (2.6)	2 (0.2)	22 (2.4)	4 (16.7)	20 (83.3)
C4.1. Duration of therapy too short	12 (1.3)	1 (0.1)	11 (1.2)	0 (0)	12 (100)
C4.2. Duration of therapy too long	12 (1.3)	1 (0.1)	11 (1.2)	4 (33.3)	8 (66.7)
C.6 medication administration process	18 (2)	9 (1)	9 (1)	1 (5.6)	17 (94.4)
C6.1. Inappropriate timing of administration and/or dosing intervals by healthcare provider	11 (1.3)	5 (0.6)	6 (0.7)	0 (0)	11 (100)
C6.2. Drug under-administered by healthcare provider	2 (0.2)	2 (0.2)	0 (0)	0 (0)	2 (100)
C6.3. Drug over-administered by healthcare provider	3 (0.3)	1 (0.1)	2 (0.2)	1 (33.3)	2 (66.7)
C6.4. Drug not administered at all by healthcare provider	2 (0.2)	1 (0.1)	1 (0.1)	0 (0)	2 (100)
C.7 patient-related factors	33 (3.6)	30 (3.3)	3 (0.3)	0 (0)	33 (100)
C7.1. Patient intentionally (knowingly) takes less or no medication than prescribed	5 (0.5)	4 (0.4)	1 (0.1)	0 (0)	5 (100)
C7.2. Patient takes more medication than prescribed	3 (0.3)	3 (0.3)	0	0 (0)	3 (100)
C7.6. Medication stored under inappropriate conditions by patient	10 (1.1)	9 (0.9)	1 (0.1)	0 (0)	10 (100)
C7.7. Inappropriate timing or dosing intervals	8 (0.9)	8 (0.8)	0	0 (0)	8 (100)
C7.8. Patient unintentionally (unknowingly) uses or administers drug incorrectly	7 (0.8)	6 (0.6)	1 (0.1)	0 (0)	7 (100)
C.8 patient transfer-related issues	26 (2.9)	9 (1)	17 (1.9)	0 (0)	26 (100)
C8.1. Medication reconciliation problem	26 (2.9)	9 (1)	17 (1.9)	0 (0)	26 (100)
C9. Others	133 (14.5)	45 (5)	86 (9.5)	23 (17.3)	110 (82.7)
C9.1. Therapy not monitored or inappropriately monitored (including therapeutic drug monitoring)	133 (14.5)	45 (5)	86 (9.5)	23 (17.3)	110 (82.7)
C.Total	907 (100)	327 (36)	580 (64)	142 (15.7)	765 (84.3)
Classification interventions					
I1. Prescriber-level	520 (41.2)	169 (13.4)	351 (27.8)	116 (22.3)	404 (77.7)
I.1.1. Prescriber was informed only	2 (0.2)	0 (0)	2 (0.2)	1 (50)	1 (50)
I1.2. Information was obtained from the prescriber	77 (6.6)	22 (1.7)	55 (4.9)	9	68 (88.3)
I1.3. Intervention was suggested to the prescriber	266 (21.6)	102 (8.1)	164 (13.5)	76 (28.6)	190 (71.4)
I1.4. Intervention was discussed with the prescriber	155 (12.8)	45 (3.6)	110 (9.2)	30 (19.4)	125 (80.6)
I.2 patient-level	93 (7.32)	85 (6.7)	8 (0.6)	9 (9.7)	84 (90.3)
I.2.1. Patient counseling provided	31 (2.42)	28 (2.2)	3 (0.3)	0 (0)	31 (100)
I 2.2. Written information only	15 (1.2)	14 (1.1)	1 (0.1)	9 (60)	6 (40)
I 2.3. Patient referred to prescriber	17 (1.3)	15 (1.2)	2 (0.1)	0 (0)	17 (100)
I 2.4. Discussion held with family member/caregiver	30 (2.4)	28 (2.2)	2 (0.1)	0 (0)	30 (100)
I3. Medication-level	548 (43.48)	188 (15)	360 (28.45)	112 (20.4)	436 (79.6)
I3.1. Drug was changed to … … … … … … …	2 (0.2)	0 (0)	2 (1.2)	0 (0)	2 (100)
I3.2. Dose was changed to … … … … … … …	227 (18)	69 (5.5)	158 (12.5)	49 (21.6)	178 (78.4)
I3.3. Formulation was changed to … … … …	36 (2.9)	17 (1.4)	19 (1.5)	5 (13.9)	31 (86.1)
I3.4. Instructions for use were changed to ……	17 (1.3)	8 (0.6)	9 (0.7)	3 (17.6)	14 (82.4)
I3.5. Drug discontinued or temporarily withheld	123 (9.8)	44 (3.5)	79 (6.2)	17 (13.8)	106 (86.2)
I3.6. New drug initiated	143 (11.25)	50 (4)	93 (6.35)	11 (7.7)	132 (92.3)
I4. Another intervention or activity	100 (8)	35 (2.8)	65 (5.2)	27 (27)	73 (73)
I4.1. Other intervention	100 (8)	35 (2.8)	65 (5.2)	27 (27)	73 (73)
I.Total	1,261 (100)	477 (37.8)	784 (62.2)	264 (20.9)	997 (79.1)
Acceptance status of interventions					
A1. Intervention accepted	547 (88.2)	200 (32.2)	347 (56)	82 (15)	465 (85)
A1.1. Intervention accepted and fully implemented	545 (87.8)	200 (32.2)	345 (55.7)	82 (15)	463 (85)
A1.3. Intervention accepted but not implemented	2 (0.4)	0	2 (0.3)	0 (0)	2 (100)
A2. Intervention not accepted	73 (11.8)	19 (3.1)	54 (8.7)	9 (12.3)	64 (87.7)
A2.1. Intervention not accepted: not suitable to administer	5 (0.8)	1 (0.1)	4 (0.7)	1 (20)	4 (80)
A2.2. Intervention not accepted: consensus not reached	68 (11)	18 (3)	50 (8.0)	8 (11.8)	60 (88.2)
A.Total	620 (100)	219 (35.3)	401 (64,7)	91 (14.7)	529 (85.3)
Resolution status of MRPs					
O1.1. Problem fully resolved	540 (87)	197 (31.8)	343 (55.3)	82 (15.2)	458 (84.8)
O3. Problem not resolved	80 (13)	21 (3.4)	59 (9.5)	9 (11.2)	71 (88.8)
O3.2. Problem not resolved due to the lack of cooperation from the prescriber	3 (0.5)	0 (0)	3 (0.5)	0 (0)	3 (100)
O3.3. Problem not resolved, intervention was ineffective	1 (0.2)	0 (0)	1 (0.2)	0 (0)	1 (100)
O3.4. Problem could not or did not need to be resolved	76 (12.3)	21 (3.4)	55 (8.8)	9 (11.8)	67 (88.2)
O. Total	620 (100)	218 (35.2)	402 (64.8)	91 (14.7)	529 (85.3)

The types and causes of MRPs were also evaluated based on the hospital unit. “Treatment effectiveness” (P1), “treatment safety” (P2), “drug selection” (C1), “dose selection” (C3), and “other” (C9) causes were found to be significantly more common in ICU patients than in ward patients. Conversely, patient-related causes (C7) were significantly more prevalent among general ward patients. Comparison of MRP types and reasons by patient care unit is given in Table 7.

**TABLE 7:** Comparison of problem types and causes by patient care unit.

Type and cause of the problem	Ward (n)	ICU (n)	p*
P1	132	239	**0.001**
P2	69	144	**0.006**
P3	17	19	0.812
C1	78	159	**<0.001**
C2	15	22	0.536
C3	129	270	**0.004**
C4	2	22	0.954
C6	9	9	0.753
C7	30	3	**<0.001**
C8	9	17	0.247
C9	45	86	**0.009**

*Mann Whitney U test.

Statistically significant values are indicated in bold in the relevant tables (*p* < 0.05).

The distribution of MRP types and causes was evaluated according to age groups, and the corresponding results are presented in Table 8. Statistically significant associations between age group and MRP categories were identified particularly for drug selection (C1), dose selection (C3), and patient-related causes (C7) (*p* < 0.05). No statistically significant differences were observed between the two age groups for the remaining MRP types and domains.

**TABLE 8:** Comparison of Problem Types and patient groups regarding pediatric or adult.

Type and cause of the problem	Pediatric (n)	Adult (n)	p*
P1	54	317	0.860
P2	30	183	0.869
P3	6	30	0.585
C1	23	214	***0.037***
C2	6	31	0.691
C3	85	314	***<0.001***
C4	4	20	0.614
C6	1	17	0.073
C7	0	33	***0.015***
C8	0	26	0.041
C9	23	110	0.944

*Mann whitney U test.

Statistically significant values are indicated in bold in the relevant tables (*p* < 0.05).

In addition to MRP classification, adverse drug events (ADEs) potentially related to treatment safety were identified during routine clinical follow-up and medication review. These events were based on clinical documentation and relevant laboratory abnormalities observed in daily practice. Selected examples of such events and their corresponding suspected medications are presented in Table 9.

**TABLE 9:** Examples of observed adverse drug event**s**.

Drugs	Adverse effect (n)
Colistin	Nephrotoxicity (5) Neurotoxicity (1)
Fluconazole	Hepatotoxicity (3)
Fosfomycin	Hypokalemia (2) Hypernatremia (1)
Linezolid	Thrombocytopenia (2)
Mycophenolate mofetil	Leukopenia (3) Diarrhea (2)
Meropenem	Anemia (1)
Metoclopramide	Confusion (1)
Piperacillin and tazobactam	Increased INR (1)
Tacrolimus	Posterior reversible Encephalopathy syndrome (PRESS) (2) Visual disturbance (1)
Terlipressin	Hyponatremia (2)
Trimethoprim-sulfamethoxazole	Hepatotoxicity (4) Pancytopenia (3)
Topiramate	Weight loss (1)
Valganciclovir	Pancytopenia (3)
Valproate	Hepatotoxicity (2)

## 4 Discussion

### 4.1 Demographic and clinical characteristics of the patients

In this study, 62.2% of the included patients were male. In the literature, similar gender distributions have been reported, with male rates of 60.9% by Murder et al., 75.9% by Pinheiro et al., and 57.8% by Shawaqfeh et al. (Mulder et al., 2021; Pinheiro et al., 2020; Shawaqfeh et al., 2023). These findings align with the literature, indicating that liver transplantation is more commonly performed in male patients. The median age of all patients in this study was 50 years (IQR: 35–61). Regarding the ages of liver transplant recipients, Schuh et al. reported a median age of 59.7 years in their study, while Mulder et al. found a median age of 59.5 years (IQR: 47–66) (Yang et al., 2019; Mulder et al., 2022). These international studies reported consistent median age values among liver transplant recipients. The comparatively lower median age in this cohort is likely attributable to the inclusion of pediatric patients who were hospitalized in the ward or ICU. When evaluating the relationship between patients’ demographic characteristics and the incidence of medication-related problems (MRPs), no significant relationship was found between gender or age and MRP occurrence. Similar results were also reported in the literature (Mulder et al., 2021; Martin and Zavala, 2004; Azadi et al., 2022).

The median number of medications used by patients in this study was 15 (IQR: 12–19; range: 8–30). In a study carried out on solid organ transplant (SOT) recipients, the mean number of medications used was reported to be 10.9 ± 3.9 (20). Another study investigating the effects of polypharmacy on transplant recipients revealed that, in the early post-transplant period, an average of 8 medications were prescribed, with the number increasing to 10–15 due to chronic comorbidities (Kim et al., 2023). These results are consistent with the result of present study. Furthermore, the total number of medications was significantly higher in the group with MRPs (*p* < 0.05). Similarly, O’Grady et al. reported that polypharmacy may reduce medication adherence and contribute to the development of MRPs (O'Grady et al., 2010). This may be due to the complex and demanding therapeutic regimens that transplant recipients must adhere to throughout their lives to treat or prevent complications.

The most frequently used drug classes among the study population were immunosuppressive agents (100%), proton pump inhibitors (99.5%), and antimicrobial agents (99.5%). In a study conducted by Duwez et al., the most commonly used medications were also immunosuppressants, followed by antimicrobials (Duwez et al., 2020). Consistent with the results achieved in the present study, Yang et al. reported that the most frequently used drug classes were immunosuppressive, antimicrobial, and antihypertensive agents, respectively (Yang et al., 2019).

The most common comorbidities among the patients in this study were hypertension (45.3%), diabetes mellitus (39.9%), chronic kidney disease (35.9%), and cardiovascular diseases (26.3%). Even though Mulder et al. reported slightly different rates, the most prevalent comorbidities were also hypertension (59%), diabetes (26%), and renal diseases (11.3%) (Mulder et al., 2022). Another study conducted in 2021 revealed the most frequently observed comorbidities to be chronic kidney disease (40.6%) and diabetes (29.7%) (Mulder et al., 2021).

In this study, the presence of at least one comorbidity increased the risk of MRPs by 3.3 times. In contrast, Azadi et al. found that having more than three comorbidities increased the risk of MRPs by 1.65 times (Azadi et al., 2022). The discrepancy in risk ratios may be due to differing thresholds used to define comorbidity-related risk. Interestingly, when comorbidities were examined, patients with a history of diabetes showed a decreased risk of MRPs. This is thought to be a coincidental finding associated with the small number of patients without MRPs in this group. On the other hand, the presence of acute kidney injury increased the risk of MRPs by 15.16 times. A literature review revealed that there was no study individually assessing comorbidities as risk factors for MRPs in transplant patients. The observed association between MRPs and acute kidney injury likely reflects overall disease severity and clinical complexity rather than a direct causal relationship.

The higher MRP burden observed among ICU patients and those with renal dysfunction suggests the presence of confounding by indication, as greater disease severity necessitates more complex pharmacotherapy and is independently associated with adverse outcomes.

### 4.2 Classification of medication related problems

In the literature, the number of studies in the literature evaluating MRPs in solid organ transplantation (SOT), including liver transplantation, is limited.

In this study, at least one MRP was identified in 311 patients (83.37%) who were admitted to either the ICU or inpatient wards. Consistent with these findings, Mulder et al. reported MRPs in 87.8% of patients, while Bonkowski et al. detected MRPs in 83.8% of SOT recipients (Mulder et al., 2022; Bonkowski et al., 2014). Although the reported incidence rates vary slightly across studies, the literature consistently demonstrates a high prevalence of MRPs in liver transplant recipients.

In this study, the most frequently observed type of MRP fell under the category of “treatment effectiveness” (P1), accounting for 59.8% of all problems, followed by “treatment safety” (P2), at 34.4%. Similarly, in a study that evaluated MRPs in SOT recipients monitored in the ICU due to COVID-19 using the PCNE classification system, “treatment effectiveness” was also the most common MRP category, comprising 34.12% of all problems, followed by “treatment safety” at 33.02% (Azadi et al., 2022). Since the MRPs in other studies involving similar patient populations were not classified according to the PCNE system, direct comparisons by problem type are not feasible. A review of the literature indicates that this study is the first and most comprehensive to classify MRPs using the PCNE v9.1 system in liver transplant recipients hospitalized in both the ICU and inpatient wards.

When focusing solely on liver transplant recipients in the ICU, the most frequently observed MRPs were again related with “treatment effectiveness” (P1), accounting for 38.5%, followed by “treatment safety” (P2), at 23.3%. In line with the findings achieved in this study, Li et al. also reported that “treatment effectiveness” (P1) was the most common category, at 41%, in ICU patients (Li et al., 2020). In other studies, evaluating MRPs using the PCNE v9.1 system, issues classified under “treatment effectiveness” (P1) became the most common category, accounting for 47.9% and 50.4% of all MRPs (Kara et al., 2024; Çakır et al., 2024). However, a study utilizing PCNE v9.0 in ICU patients reported that 43.4% of MRPs were related with “treatment safety” (P2), and another similar study found this rate to be as high as 77.18% (Durmuş et al., 2024; Albayrak et al., 2022). These variations suggest that differences in the most frequently encountered MRP categories may be attributable to differences in patient populations across studies.

A review of the literature reveals no existing studies investigating MRPs associated with renal dose adjustment in liver transplant recipients. In this study, among ICU patients with renal impairment who required renal dose adjustments, 24.3% had at least one MRP related to these medications. Notably, these problems accounted for the majority of those categorized under “treatment safety” (P2). In a study carried out by Garin et al., the incidence of MRPs associated with renal dose adjustments in ICU patients was reported as 17%, while Alsayed et al. reported a rate of 22% (Garin et al., 2021; Ali et al., 2024).

In this study, the most frequently encountered cause of MRPs was “dose selection” (C3), accounting for 44.2% of cases, followed by “drug selection” (C1) at 26.1%. Similarly, in a study carried out by Repp et al. among heart transplant patients, “dose selection” was reported as the most common cause of MRPs at 48.2%. This was followed by Duwez et al.’s study in lung transplant recipients (39.6%) and Lee et al.’s study in kidney transplant recipients (38%) (Bonkowski et al., 2014; Lima et al., 2016; Lee et al., 2016). These findings suggest that clinical pharmacists’ recommendations regarding dose selection can significantly contribute to the prevention of MRPs.

When evaluating MRP causes identified exclusively in critically ill patients, the most frequent issue was again found to be related with “dose selection” (C3), accounting for 30% of cases, consistent with the overall findings of this study. In a study carried out by Kara et al., “dose selection” was also the most frequently observed MRP cause, reported at 77% (Kara et al., 2024).

Within the category of MRPs classified under “drug selection,” the most common subcategory was “absence of drug therapy despite an existing indication” (C1.5), which accounted for 17.8% of cases. This aligns with the findings achieved by Mulder et al., who reported an untreated indication rate of 15%, and those achieved by Wang et al., who found this rate to be 20.9% in a study on kidney transplant recipients (Mulder et al., 2022; Wang et al., 2008).

In this study, the most frequently observed subcategory under the “other” classification was “treatment outcome not monitored or monitored inappropriately (including therapeutic drug monitoring)” (C9.1), at 14.4%. In line with these results, Duwez et al. reported a frequency of 11.2% for this issue, Covert et al. reported 13% in kidney transplant patients, and Yang et al. found a rate of 10% in their study on kidney transplant recipients (Duwez et al., 2020; Yang et al., 2019; Covert et al., 2017).

A total of 620 suggestions were offered for the 620 identified MRPs in this study. The majority of these suggestions were directed at the “drug level” (I3) (43.45%) and at the “prescribing physician level” (I1) (41.2%).

Among the suggestions related with MRPs at the “drug level” (I3), the most common suggestion was “dose changed to…” (I3.2), which accounted for 18% of the suggestions. Consistent with these findings, Pinheiro et al. reported a similar rate of 18.2% for dose change suggestions, Wang et al. reported 15%, and Lima et al. found a rate of 10.2% (Pinheiro et al., 2020; Wang et al., 2008; Lima et al., 2016). In a study carried out by Duwez et al., change recommendations constituted 43.8% of all interventions, while this proportion was 33.8% in the study carried out by Mulder et al. (Bonkowski et al., 2014; Kara et al., 2024). These variations in reported frequencies are likely attributable to differences in the methods used for MRP classification.

Considering the “drug level” (I3) category, the second most common suggestion was “initiation of a new medication” (I3.6), accounting for 11.2% of the suggestions. In line with these results, Mulder et al. reported a rate of 10.2% for similar suggestions, and Pinheiro et al. reported 11% (Mulder et al., 2021). However, in the study carried out by Azadi et al., “initiation of a new medication” (I3.6) was reported at a significantly lower rate of 1.83% (Azadi et al., 2022). The discrepancy among studies may be due to additional suggestions related to patients’ therapeutic regimens during the COVID-19 pandemic.

Of the suggestions offered for MRPs identified in this study, 88.2% were accepted. This acceptance rate is comparable to those reported in the literature, such as 93% reported by Lee et al. for kidney transplant recipients, 93.6% by Mulder et al., and 95% in another study involving both kidney and liver transplant recipients (Pinheiro et al., 2020; Mulder et al., 2022; Lee et al., 2016). These findings highlight the high acceptance rates of clinical pharmacists’ suggestions worldwide in promoting safe and effective pharmacotherapy.

Although the PCNE v9.1 classification was employed as a standardized framework for the systematic categorization of MRPs, its standalone presentation may be perceived as largely descriptive if not accompanied by clinical context. To enhance clinical interpretability, a Supplementary Table S1 has been added, providing representative, real-word clinical examples from our cohort. This table illustrates the application of the PCNE v9.1 classification across the problem, cause, intervention and outcome domains, thereby contextualizing the classification within routine clinical practice.

The ADEs observed in the present study illustrate key pharmacotherapy-related safety risks inherent to liver transplantation. Predominant renal, hematological, neurological, and electrolyte disturbances associated with commonly used immunosuppressive and antimicrobial agents highlight the narrow therapeutic margins and cumulative toxicity burden in this population. These findings provide clinically relevant safety context for MRPs and underscore the importance of systematic medication review and close pharmacotherapy monitoring in routine transplant care.

In the evaluation of MRPs, particularly in highly complex patient populations, a delicate balance exists between the standardized recommendations provided by clinical practice guidelines and individualized, patient-specific clinical decision-making processes Although clinical practice guidelines aim to standardize medical care based on scientific evidence, treatment decisions are often influenced by the patient’s clinical condition, comorbidities and broader contextual factors (Mercuri et al., 2015). In this context, some of the MRPs identified in the present study may reflect deliberate deviations from guideline recommendations driven by individualized clinical judgment in specific clinical scenarios, rather than in appropriate treatment practices *per se*. From a clinical pharmacist perspective, such deviations were classified as MRPs due to their potential risk profile. Moreover, the non-acceptance of certain pharmacist recommendations by the clinical team may represent patient-spesific,context-driven decision-making rather than erroneous clinical practice. Taken together, these conditions suggest that the concept of MRPs should not be viewed solely as indicators of absolute inappropriateness, but rather within a broader framework that also encompasses clinical risk awareness and the need for multidisciplinary evaluation.

### 4.3 Strengths and limitations

Among the strengths of this study are its prospective design, which allowed for direct communication with both patients and healthcare professionals, and its inclusion of the entire transplantation unit. To the best of our knowledge, this is the first prospective study in which all liver transplant recipients admitted to both the ICU and inpatient ward were included, medication-related problems (MRPs) were identified by a clinical pharmacist, suggestions were offered, and all findings were classified using the PCNE v9.1 system.

This study has several limitations. First, its single-center design may limit the generalizability of the findings, and the absence of a control group precludes causal inferences regarding the impact of clinical pharmacy interventions. In addition, no structured educational training program was provided to patients or healthcare staff as part of the study. Although MRPs and pharmacist interventions were prospectively identified and classified, the clinical severity and potential harm associated with individual MRPs were not systematically assessed. Furthermore, patient-centered clinical outcomes following pharmacist recommendations were not evaluated in a structured manner. As the primary aim of the study was to characterize MRPs and pharmacist interventions within a highly complex transplant population, outcome-oriented analyses were beyond the scope of the present investigation.

No formal *a priori* sample size or power calculation was performed, as the study was designed as a prospective observational investigation including all eligible liver transplant recipients admitted during the predefined study period. Given the limited sample size, subgroup analyses based on key transplant-related factors-such as immunosuppressive burden, renal function, and infection status were not performed, as could have result in small subgroup sizes and increased the risk of model overfitting, thereby compromising the robustness of effect estimates. In addition, despite the use of a conservative variable selection strategy to construct a parsimonious multivariable model, the limited number of events for certain predictors particularly AKI – may have contributed to wide confidence intervals and potential sparse-data bias, warranting cautious interpretation of these findings. Collectively, these limitations may restrict the clinical interpretability of the findings.

Future research should focus on outcome-oriented study designs incorporating validated severity grading of MRPs, structured assessment of patient-centered clinical outcomes (e.g., renal function, infection rates, or length of hospital stay), and appropriate control groups. In addition, adequately powered multicenter studies enabling stratified and subgroup analyses based on key transplant – related factors may further clarify the clinical impact and added value of pharmacist-led interventions in transplant care.

### 4.4 Conclusion

The literature review suggests that this study is the first and most comprehensive investigation to classify MRPs in liver transplant recipients hospitalized in both the ICU and the inpatient ward using the PCNE v9.1 methodology. Furthermore, it is the first study to demonstrate differences in MRP categories between patients in the ICU and those in the general ward. The most common MRP categories identified in both ICU and ward patients were “treatment effectiveness,” with the primary causes being “inappropriate dose selection” and “inappropriate drug selection.

This study is that MRPs in liver transplant recipients frequently reflect the complexity of individualized clinical decision-making rather than isolated prescribing errors. By identifying and contextualizing these risks, clinical pharmacists support rational pharmacotherapy and informed multidisciplinary decision-making in complex transplant care.

The presence of at least one comorbidity and acute kidney injury was found to be an independent risk factors for MRPs in liver transplant recipients, contributing novel data to the existing body of knowledge.

This study concludes that clinicians should exercise particular caution when prescribing new medications to liver transplant recipients with comorbid conditions and a history of acute kidney injury.

Both clinical pharmacists and clinicians should pay close attention to “dose selection” and “drug selection” when reviewing the treatment plans of patients in liver transplant units.

Moreover, this study demonstrates that the active involvement of clinical pharmacists as part of the multidisciplinary team significantly contributes to the successful management of MRPs in transplant recipients with complex medication regimens.

## Data Availability

The original contributions presented in the study are included in the article/Supplementary Material, further inquiries can be directed to the corresponding author.
