# Supplementary material for: Prospective evaluation of medication-related problems and pharmacist interventions in liver transplant recipients
Source: Front Pharmacol. 2026 Feb 23;17:1738563. doi: 10.3389/fphar.2026.1738563 (PMC12968275; doi:10.3389/fphar.2026.1738563)
Supplement: Supplementary file 1 [file Table1.docx]

**Supplementary Table 1: Example of PCNE V9.1 Problem Categories with Clinical Scenarios**

| **Type of MRP** | **Example** |
| --- | --- |
| **P1. Treatment Effectiveness** | |
| **P1.2.** Drug therapy not sufficiently effective | Early post-transplant administration of hepatitis B immunoglobulin as monotherapy. |
| **P1.3.** There are untreated symptoms or indications | Absence of corticosteroid therapy in the maintenance treatment regimen of patient who underwent liver transplantation for primary sclerosing cholangitis. |
| **P2.1. Treatment safety** | Increase in international normalized ratio (INR) associated with piperacillin-tazobactam use. |
| **P3. Others** | |
| **P3.1.** Unnecessary drug therapy | Administration of piracetam therapy despite the absence of cognitive impairment in the patient. |
| **Causes of MRP** | **Example** |
| **C1. Drug Selection** | |
| **C1.1.** Drug not appropriate per guidelines/formulary | Use of tigecycline as first-line antibiotic therapy in a patient with urinary tract infection |
| **C1.2.** No indication for the drug | Administration of antidiabetic therapy to patient without a diagnosis of diabetes or related comorbidities. |
| **C1.3.** Inappropriate combination with other drugs and/or supplements such as herbal ones | Increase in tacrolimus blood level associated with concomitant use of tigecycline. |
| **C1.4.** Unnecessary duplication within drug class or active ingredient | Concomitant prescription of two different pharmaceutical products containing ipratropium bromide in the same patient. |
| **C1.5.** Drug not prescribed for an existing indication | Failure to administer Cytomegalovirus (CMV) prophylaxis with valganciclovir/ganciclovir despite a clear indication in the patient. |
| **C1.6** Excessive variety of drugs for same indication | A combined treatment approach involving the concomitant administration of norepinephrine and terlipressin with albumin support in an intensive care patient who developed hepatorenal syndrome. |
| **C2 Dosage Form** | |
| **C2.1.** Inappropriate dosage form/formulation for the patient | Use of extended-release metoprolol succinate in a patient receiving enteral tube feeding. |
| **C3. Dosage selection** | |
| **C3.1.** Drug dose too low | In a pediatric patient with Wilson disease weighing 30 kg, trientine was prescribed at a daily dose of 250 mg, which falls below the recommended dosing range of 10-15 mg/kg/day. |
| **C3.2.** Drug dose too high | In a patient with reduced renal function (eGFR:50 mL/dk 1.73 m^2^), digoxin was prescribed at 0.25 mg/day instead of the recommended 0.125 mg/day. |
| **C3.3.** Dosing regimen not frequent enough | In a patient with impaired renal function, teicoplanin was administered at 72-hour intervals instead of the recommended 48-hour dosing schedule, resulting in an extended dosing interval. |

| **Supplementary Table 1: Example of PCNE V9.1 Problem Categories with Clinical Scenarios (Continue)** | |
| --- | --- |
| **C3.4.** Dosing regimen too frequent | In a patient with impaired renal function, meropenem was administered every 8 hours rather than the recommended 12-hour interval, resulting in increased dosing frequency. |
| **C3.5.** Instructions regarding timing of dose are unclear, incorrect, or incomplete | Administration of corticosteroids in the evening rather than in the morning, contrary to dosing aligned with the patient’s circadian rhythm. |
| **C4.** **Duration of Therapy** |  |
| **C4.1.** Duration of therapy too short | Discontinuation of mycophenolate mofetil in the patient before completion of the recommended minimum 3-month treatment period. |
| **C4.2.** Duration of therapy too long | Administration of terlipressin for 10 days in a patient with gastroesophageal variceal bleeding, exceeding guideline-recommend duration. |
| **C.6 Medication Administration Process** | |
| **C6.1.** Inappropriate timing of administration and/or dosing intervals by healthcare provider | Ceftazidime-avibactam was administered as a 30-minute infusion instead of the recommended 2-hour infusion required to achieve pharmacokinetic/pharmacodynamic targets. |
| **C6.2.** Drug under-administered by healthcare provider | Levothyroxine was taken by the patient in the evening,contrary to the recommended morning fasting administration. |
| **C6.3.** Drug over-administered by healthcare provider | In a patient requiring dose reduction based on impaired hepatic function as defined by the Child-Pugh score, tigecycline was administered at 100 mg/day instead of the recommended 50 mg/day. |
| **C6.4.** Drug not administered at all by healthcare provider | Failure to administer Vitamin E supplementation prescribed for the treatment of non-alcoholic fatty liver disease (NAFLD) in the patient. |
| **C.7 Patient-Related Factors** | |
| **C7.1.** Patient intentionally (knowingly) takes less or no medication than prescribed | The patient independently discontinued insulin therapy without the knowledge or recommendation of the treating physician. |
| **C7.2.** Patient takes more medication than prescribed | The patient took escitalopram two times daily despite a prescription for one-daily dosing |
| **C7.6.** Medication stored under inappropriate conditions by patient | Trientine capsules were stored at room temperature by the patient despite the label-recommended storage conditions. |
| **C7.7.** Inappropriate timing or dosing intervals | Administration of trimethoprim-sulfamethoxazole without adherence to the appropriate dosing intervals. |
| **C7.8.** Patient unintentionally (unknowingly) uses or administers drug incorrectly | Failure of the patient to adhere to the recommended dosing timing of tacrolimus, namely, administration at least 30 minutes before meals or 2 hours after meals. |
| **C.8** Patient transfer-related issues | |
| **C8.1.** Medication reconciliation problem | Despite a history of chronic hypertension treated with amlodipine 5 mg/day, this medication was not prescribed during hospitalization. |
| **C9. Others** | |
| **Supplementary Table 1: Example of PCNE V9.1 Problem Categories with Clinical Scenarios (Continue)** | |
| **C9.1.** Therapy not monitored or inappropriately monitored (including therapeutic drug monitoring) | Despite tacrolimus blood concentrations being outside the therapeutic range, no dose adjustment was made in the patient’s treatment. |
| **Classification Interventions** | **Example** |
| **I1.** Prescriber-level |  |
| **I.1.1.** Prescriber was informed only | The HbsAg and anti-HBs serological results of the patient receiving hepatitis B immunoglobulin therapy were communicated to the attending physician. |
| **I1.2.** Information was obtained from the prescriber | Information regarding the patient’s status with respect to portal vein thrombosis was obtained through consultation with the attending physician. |
| **I1.3.** Intervention was suggested to the prescriber | A dose adjustment recommendation for tigecycline, taking hepatic function into consideration, was communicated to the attending physician. |
| **I1.4.** Intervention was discussed with the prescriber | The use of idebenone therapy was discussed in a patient who developed visual impairment associated with tacrolimus treatment. |
| **I2. Patient-level** | |
| **I2.1.** Patient counseling provided | Patients were provided with counseling on medication use, including drugs requiring specific administration skills, such as inhaler techniques, subcutaneous insulin injections. |
| **I2.2.** Written information only | Written information was provided to the liver transplant patient receiving immunosuppressive therapy stating that live vaccines are contraindicated and that vaccination planning should be carried out according to the physician's recommendation. |
| **I2.3.** Patient referred to prescriber | The patient with suspected metformin-related vomiting and diarrhea was referred to the endocrinology clinic. |
| **I 2.4.** Discussion held with family member/caregiver | The caregiver was informed about the storage conditions of insulin pens both before first use and after initiation of use. |
| **I3. Medication-level** | |
| **I3.1.** Drug was changed to ………………… | In a patient receiving tacrolimus and tigecycline, an increase in tacrolimus levels was observed; therefore, based on our recommendation, tigecycline was replaced with colistin. |
| **I3.2.** Dose was changed to ………………… | The patient’s everolimus dose was increased based on the results of therapeutic drug monitoring. |
| **I3.3.** Formulation was changed to …………. | The patient was switched from prolonged-release tacrolimus to immediate-release tacrolimus in order to achieve a more rapid increase in blood concentrations. |
| **I3.4.** Instructions for use were changed to …. | The tacrolimus regimen was adjusted to be administered after interruption of enteral feeding. |
| **I3.5.** Drug discontinued or temporarily withheld | Mycophenolate mofetil therapy was discontinued in patients with a white blood cell count below 3,000/ µL |

| **Supplementary Table 1: Example of PCNE V9.1 Problem Categories with Clinical Scenarios (Continue)** | |
| --- | --- |
| **I3.6.** New drug initiated | Ursodeoxycholic acid therapy was initiated in patients who underwent liver transplantation for primary sclerosing cholangitis. |
| **I4. Another intervention or activity** |  |
| **I4.1.** Other intervention | Providing information to the healthcare team regarding drug-food interactions or interactions between medications and enteral/parenteral nutrition. |
| **Acceptance Status of Interventions** | **Example** |
| **A1. Intervention accepted** |  |
| **A1.1.** Intervention accepted and fully implemented | Interruption of everolimus therapy was recommended following the development of nephrotic-range proteinuria (≥ 3g/day) |
| **A1.3.** Intervention accepted but not implemented | Initiation of intravenous immunoglobulin (IVIG) therapy was planned following the detection of thrombocytopenia; however, the treatment could not be implemented due to the patient’s death. |
| **A2. Intervention not accepted** | |
| **A2.1.** Intervention not accepted: not suitable to administer | Although signs of rejection were observed in the patient, no intervention to increase tacrolimus blood levels was undertaken due to the presence of sepsis. |
| **A2.2.** Intervention not accepted: consensus not reached | In the pediatric patient,enoxaparin at a dose 0,5 mg/kg every 12 hours was recommended for venous thromboembolism prophylaxis; however, the recommendation was not accepted; and treatment was continued at 0,5 mg/kg once daily. |
| **Resolution Status of MRPs** | **Example** |
| **O1. Problem totally solved** | |
| **O1.1.** Problem fully resolved | In the context of stress ulcer prophylaxis, adjustment of pantoprazole dosing from 40 mg twice daily to 40 mg every once daily was recommended. |
| **O3. Problem not resolved** | |
| **O3.2.** Problem not resolved due to the lack of cooperation from the prescriber | Despite inadequate caloric intake via enteral nutrition, total parenteral nutrition was not initiated following clinical evaluation. |
| **O3.3**. Problem not resolved, intervention was ineffective | IVIG was recommended and accepted due to thrombocytopenia; however, its effectiveness could not be evaluated because of the patient’s death. |
| **O3.4.** Problem could not or did not need to be resolved | In a patient with a CMV viral load of 100 copies, valganciclovir therapy was not considered indicated. |
